# Epidemiology and audiologic characteristics of neonatal hearing loss: A cross-sectional study

**DOI:** 10.1371/journal.pone.0332612

**Published:** 2025-09-30

**Authors:** Maojie Liu, Qiuying Xie, Xi He, Qiuya Jiang, Dan Lai

**Affiliations:** Department of Otolaryngology Head and Neck Surgery, The Affiliated Hospital of Southwest Medical University, Luzhou, China; Center for Healthy Start Initiative, NIGERIA

## Abstract

**Objective:**

To explore the epidemiology and audiologic characteristics of neonatal hearing loss in neonates in southwestern China during 2020–2023.

**Methods:**

This cross-sectional study involved all neonates born in Luzhou (China) during 2020–2023. Neonates with hearing loss (HL) were identified through initial hearing screening, rescreening, and diagnostic hearing test, and risk factors were determined using a questionnaire.

**Results:**

From 2020 to 2023, there were a total of 109,562 neonates in Luzhou. Among them, 286 (465 ears) newborns were diagnosed with HL, with an incidence of 2.61 per thousand (2.61‰) (2020: 2.06‰, 2021: 2.89‰, 2022: 2.68 ‰, 2023: 3.00‰), and the difference in incidence between years was not statistically significant (P = 0.104). Among the affected ears, 234 (1.07‰) had mild HL, 130 (0.59‰) had moderate HL, 30 (0.14‰) had severe HL, and 71 (0.32‰) had profound HL. Additionally, 104 (0.95‰) children had unilateral hearing loss (UHL), while 182 (1.66‰) children had bilateral hearing loss (BHL), the incidence of UHL and BHL did not significantly differ across years (P = 0.33). There were 171 male children (2.99‰) and 115 female children (2.19‰) who were diagnosed with HL, and no significant difference was found in incidence between sexes (P = 0.10). The cause of HL remained unknown in 94 (32.87%) children, while 192 (67.13%) children had identifiable risk factors, including neonatal jaundice (93 cases), neonatal ward history (90 cases), pregnancy history (85 cases), and a family history of HL (24 cases). Of these, 113 (39.51%) children had multiple risk factors, while 79 (27.62%) children had only one identified risk factor.

**Conclusion:**

During 2020–2023, the incidence of neonatal hearing loss is 2.61‰, which is not significantly increase, and no significant abnormal factors were identified.

## Introduction

Neonatal hearing loss is defined as hearing loss (HL) presenting at birth, arising from disturbances in sound conduction and/or sound conversion into neural electrical impulses, making it as one of the most common sensory disorders [1]. Children with HL present delay in language learning and general development, which can only be prevented by early diagnosis and management [2]. The prevalence of HL has globally exhibited an upward trend [3]. According to the World Health Organization (WHO) report published in 2021, 340,000 children suffered from neonatal HL globally [4]. Before the emergence of universal newborn hearing screening (UNHS), neonatal HL was diagnosed at an average age of 2–3 years [5]. However, the comprehensive audiologic evaluation can be currently completed by 3 months [6].

It has been reported that the incidence of hearing loss in newborns is about 1–3 per thousand (1–3‰) [7], although its incidence varies across countries and regions. In the United States, the average incidence was estimated at 1.1 per 1,000 infants, with state-specific variations ranging from 0.22 to 3.61 per 1,000 [8]. In Japan, the prevalence of neonatal HL has been reported at 0.14% [9]. A meta-analysis of 48 studies found that the mean prevalence of HL among newborns was 5‰ in India and 2‰ in other Southeast Asian countries, including Thailand, Malaysia, and Nepa [10]. The etiology of neonatal HL is complex and may be associated with genetic susceptibility, intrauterine infection, and perinatal factors [11]. The etiology of neonatal HL is complex and may be caused by genetic susceptibility, intrauterine infection, and perinatal factors. According to the American Federation of Infant Hearing (JCIH), up to 13 risk factors have been identified [6], while multiple studies have reported the existence of numerous unexplained factors [12]. Studies found that the number of neonatal risk factors is growing, and the likelihood of hearing impairment also increases [13]. The prevalence of hearing impairment in infants with risk factors has been reported at 8–13‰ in India [14], reaching as high as 17% in very low birth weight infants [15]. Notably, the incidence of neonatal HL among infants admitted to the neonatal intensive care unit (NICU) is significantly higher (2.73‰) compared with that in normal newborns (1.98‰) [16]. Due to the high prevalence of HL in the NICU, hearing screening is recommended for all infants admitted to the NICU.

According to the China Disabled Persons’ Federation, an estimated 20,000–30,000 newborns with HL are born in China each year [17]. The incidence of neonatal HL in China ranges from 1.66‰ to 3.26‰ [16,18–20], and there is no significant difference in its incidence particularly in rural and urban areas [16]. However, due to China’s vast population and regional disparities in economic development and healthcare access, the incidence of neonatal HL varies across different regions, with reported rates of 2.8‰ in the eastern region, 2.2‰ in the central region, and 1.7‰ in the western region [21]. Luzhou, located in southwest China, is a densely populated area with a relatively underdeveloped economy and a diverse population that includes multiple ethnic minorities. Despite these demographic and socioeconomic factors, comprehensive epidemiological studies on neonatal HL in this region remain scarce. Therefore, this study aimed to assess whether the incidence and risk factors for neonatal HL exhibit significant variability in southwest China. Additionally, the study examined key epidemiological characteristics, including the incidence of BHL and UHL, sex-based differences, and variations in HL severity. The findings may provide an epidemiological foundation for developing targeted prevention and treatment strategies for hearing impairment in economically underdeveloped regions of western China.

## Methods

### 1. Study population

This cross-sectional study retrospectively analyzed data from all live births in 86 midwifery institutions across four counties and three districts of Luzhou between January 1, 2020, and December 31, 2023. All children with hearing loss underwent both diagnostic hearing test and survey at the Luzhou Sub-center for the Diagnosis and Treatment of Neonatal Hearing Impairment in the Affiliated Hospital of Southwest Medical University (Luzhou, Sichuan Province, China). The survey data were obtained from the annual report of Luzhou Maternal and Child Newborn Screening Management Office and the confirmed diagnosis information provided by the Luzhou Sub-center for the Diagnosis and Treatment of Neonatal Hearing Impairment in the Affiliated Hospital of Southwest Medical University.

### 2. Ethical requirement

This study was carried out in accordance with the Declaration of Helsinki and approved by the ethics committee of the hospital (Approval No. KY2024082). The screening procedure was conducted in accordance with the technical specifications for universal hearing screening issued by the Chinese Ministry of Health. Infants’ parents signed the informed consent form prior to hearing screening.

### 3. Hearing screening and diagnostic procedure

The hearing screening organizations in Luzhou followed the requirements of the former Ministry of Health’s Technical Specifications for Newborn Hearing Screening (2010 Edition) [22]. Initial hearing screening was generally performed at the maternity hospital where the child was born or at the local Maternal and Child Health Center (MCH) with hearing screening qualifications from 48 h after birth until discharge from the hospital. It was essential to ensure that all newborns were in a separate, well-ventilated, quiet (ambient noise ≤ 45 dBA) room at the time of hearing screening, the infant’s external auditory canal secretions were cleared prior to screening, and the screening was carried out while the baby was in its natural sleep state. Transient evoked otoacoustic emission (TEOAE) or distortion product otoacoustic emission (DPOAE) was utilized for the initial hearing screening and rescreening for newborns with normal delivery. The automated auditory brainstem response (AABR) was employed for screening newborns who were in NICUs.

Participants who showed abnormal results during the initial hearing screening or missed the screening were rescreened bilaterally at the same hearing screening unit or a designated hospital within 42 days. OAE or AABR is typically used for the rescreening, and some screening organizations use their combination. If any of the rescreening results were abnormal, diagnostic hearing tests were performed.

Those who failed in the rescreening were referred to the Luzhou Sub-center for the Diagnosis and Treatment of Neonatal Hearing Impairment in the Affiliated Hospital of Southwest Medical University within 3–6 months after birth for diagnostic hearing tests. Diagnostic tests, including acoustic immittance (probe sound, 226 and 1000 HZ), ABR, auditory steady state response (ASSR), OAE, and imaging tests, if necessary, were utilized for cross-validation to fully assess hearing function in infants and young children. The average hearing thresholds of 500, 1000, 2000, and 4000 Hz were utilized to classify neonatal HL and hearing impairment as follows: mild (26–40 dB HL), moderate (41–60 dB HL), severe (61–80 dB HL) and profound (≥81 dB HL) [23].

### 4. Questionnaire

All newborns with HL underwent survey, involving a standardized questionnaire that was developed by the Sichuan Hearing Impairment Diagnosis and Treatment Center (Appendix A). A professional audiologist filled the questionnaire, including demographic characteristics and risk factors. Based on the risk factors reported by JCIH [6] combined with local characteristics, the following risk factors were counted and analyzed in this study, including pregnancy history, low birth weight (<2,500g), gestational age < 28 weeks, history of hypoxia, neonatal jaundice, neonatal ward history, history of neonatal disease, family history of HL, craniofacial anomalies, newborn or mother’s treatment with ototoxic drugs, and other suspicious factors. Regarding pregnancy and neonatal history, we registered and analyzed the risk factors for neonatal hearing loss based on the clinical diagnosis of outpatient cases, inpatient cases, and discharge certificates, and the genetic history was collected based on the detailed family history provided by the family members, and all positive family histories of hearing loss were registered and analyzed.

### 5. Statistical analysis

Data were analyzed using SPSS 27.0 software (IBM, Armonk, NY, USA). In descriptive analysis, demographic characteristics, audiological outcomes, and risk factors for HL were summarized using counts and percentages. The annual incidence, incidence of HL at varying severity levels, incidence by ear side, and gender-based incidence were compared using the Chi-square test. Non-parametric variables, such as risk factors and degrees of HL were analyzed by Kruskal-Wallis test (H test). P < 0.05 was considered statistically significant.

## Results

### 1. Incidence of HL in newborns

From January 1, 2020 to December 31, 2023, there were 109,562 neonates in Luzhou area. According to the neonatal hearing diagnosis and treatment database of the Luzhou Sub-center for the Diagnosis and Treatment of Neonatal Hearing Impairment in Sichuan Province, 286 (2.61‰) children were diagnosed with HL, including 69 (2.06‰) children in 2020, 80 (2.89‰) children in 2021, 68 (2.68‰) children in 2022, and 69 (3.00‰) children in 2023. The incidence of neonatal HL across various years was not statistically significant (P = 0.104) (Table 1).

**Table 1:** Incidence of neonatal HL in Luzhou during 2020-2023.

Year	Newborns (N)	HL (N)	Incidence (‰)	*χ²*	*P*
2020	33519	69	2.06	6.153	0.104
2021	27659	80	2.89		
2022	25385	68	2.68		
2023	22999	69	3.00		
Total	109562	286	2.61		

### 2. Incidence and distribution of different degrees of HL

**Fig 1:**
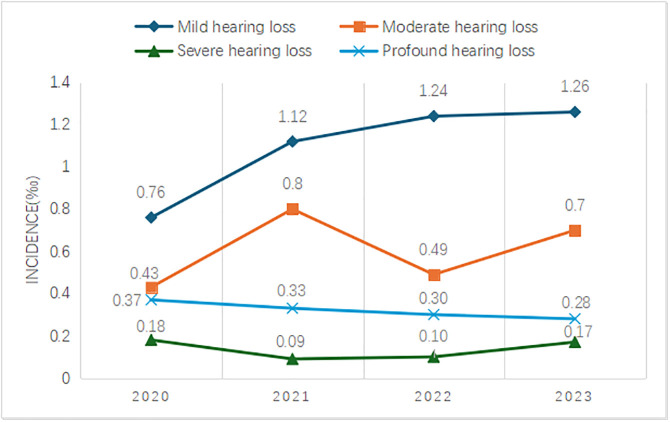
Incidence of varying degrees of HL.

From 2020 to 2023, 173,126 ears were screened, and diagnostic hearing tests were performed on 572 ears, of which 465 ears were diagnosed with varying degrees of HL. Moreover, 234 (1.07‰), 130 (0.59‰), 30 (0.14‰), and 71 (0.32‰) ears suffered from mild HL, moderate HL, severe HL, and profound HL, respectively (Fig 1). Of these 465 ears, about half had mild HL, followed by moderate HL and severe HL. Additionally, the incidence of mild HL has been steadily increasing each year, while the incidence of other degrees of HL has exhibited some fluctuations but remained relatively stable.

### 3. HL by ear side and gender

**Fig 2:**
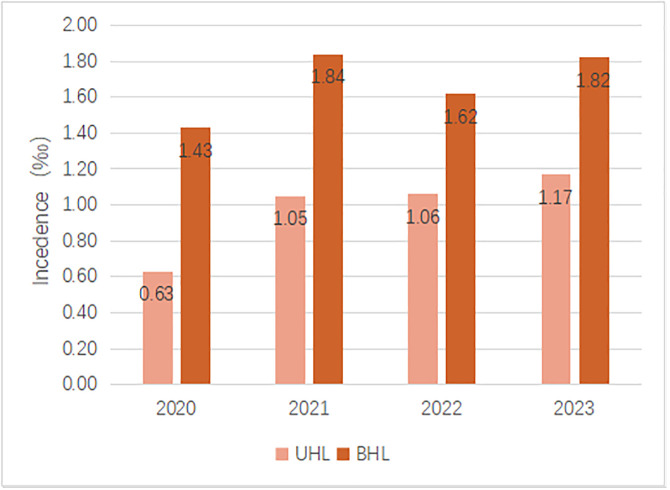
Incidence of UHL and BHL in newborns.

**Fig 3:**
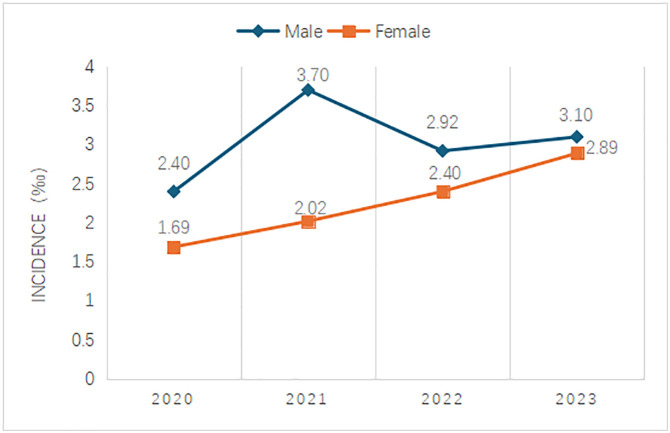
Incidence of HL in newborns with different genders.

Among 286 children with HL, 104 had UHL with an incidence of about 0.95‰ and 182 had BHL with an incidence of approximately 1.66‰, and the overall incidence of BHL was higher than that of UHL. As illustrated in Fig 3, the annual prevalence of BHL (2020: 1.43‰; 2021: 1.84‰; 2022: 1.62‰; 2023: 1.82‰) was also higher than that of UHL (2020: 0.63‰; 2021: 1.05‰; 2022: 1.06‰; 2023: 1.17‰), which were analyzed by Chi-square test. The results indicated no significant difference in the incidence of UHL and BHL across different years (P = 0.333) (Fig 2).

From 2020 to 2023, 57,146 male newborns were screened, and 171 were diagnosed with HL, with an incidence of about 2.99‰; 52,415 female newborns were screened, and 115 were diagnosed with HL, with an incidence of approximately 2.19‰. The difference in incidence of neonatal HL between male and female infants was statistically significant (P = 0.01), and the incidence was higher in male infants than that in female infants (Fig 3).

### 4. Analysis of risk factors for neonatal HL

**Fig 4:**
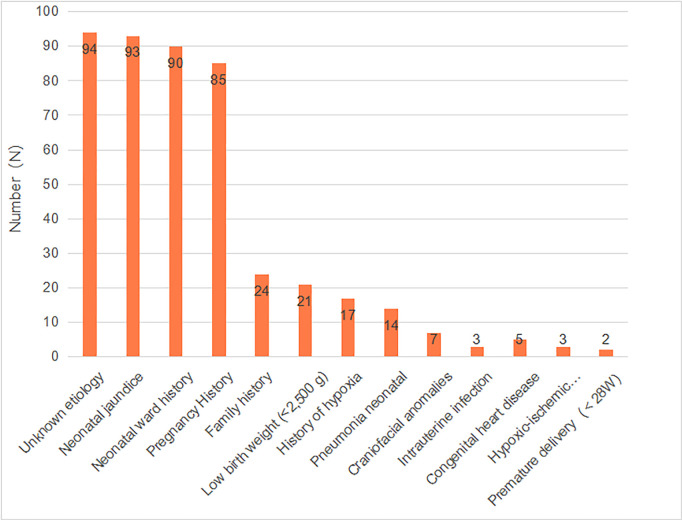
Risk factors associated with hearing loss in newborns from 2020 to 2023. Note: Multiple counts were recorded for children with HL who had multiple risk factors.

Notably, 286 newborns with neonatal HL were analyzed for medical history. Among them, 113 (39.51%) cases experienced the impact of multiple risk factors, and multiple counts were influenced by multiple risk factors, with multiple counts recorded for cases with more than one risk factor. Additionally, 79 (27.62%) cases were affected by a single risk factor, while 94 (32.87%) cases had unexplained HL. Furthermore, 192 (67.13%) cases were found to have identifiable risk factors, which included neonatal jaundice (93 cases), neonatal ward history (90 cases), pregnancy history (85 cases), family history of deafness (24 cases), low birth weight (21 cases), and premature delivery (2 cases) (Fig 4). Upon analyzing the degree of HL associated with different risk factors, it was found that most risk factors were primarily linked to mild HL, while those associated with a family history of deafness were more commonly linked to moderate and profound HL (Table 2).

**Table 2:** Risk factors associated with hearing loss in newborns from 2020 to 2023.

Risk factors	Normal	Mild	Moderate	Severe	Profound	Ears (N)
Unknown etiology	35	80	41	8	24	188
Neonatal jaundice	28	82	45	15	16	186
Neonatal ward history	35	74	41	12	18	180
Pregnancy History	31	71	41	3	24	170
Family history	7	11	16	1	13	48
Low birth weight (<2,500 g)	9	18	8	2	5	42
Pneumonia neonatal	5	12	7	0	4	28
History of hypoxia	8	13	5	5	3	34
Craniofacial anomalies	3	1	7	2	1	14
Intrauterine infection	2	1	3	0	0	6
Congenital heart disease	1	5	3	0	1	10
Hypoxic-ischemic encephalopathy	1	3	1	0	1	6
Premature delivery (<28W)	1	3	0	0	0	4

## Discussion

Neonatal HL is one of the common causes of HL in children. With the implementation of UNHS and improved perinatal care, there has been a relative increase in the recognition of neonatal HL. In the present study, the incidence of neonatal HL during 2020–2023 in Luzhou was 2.61‰, which was basically consistent with the 1–3 per 1,000 reported in previous studies [16,18–20]. The study period coincided with the COVID-19 epidemic. The present study indicated that the prevalence of neonatal HL in Luzhou did not seem to be affected by the COVID-19 epidemic and did not significantly vary. This may indirectly demonstrate that COVID-19 infection may not be a risk factor for neonatal HL.

China involves a vast territory with diverse ethnic groups and varying levels of economic, education, and health development across different regions, leading to differences in the prevalence of neonatal HL in various regions. According to available reports, in economically developed coastal areas, such as Shanghai and Guangdong, the incidence rates were 1.7‰ and 3.7‰ [21], respectively. In the less economically developed western regions, such as Yunnan and Xinjiang, the incidence rates were 2.30‰ and 2.33‰, respectively [21]. Despite this, the incidence of neonatal HL in Luzhou had remained relatively stable over the past four years (2020: 2.06‰, 2021: 2.89‰, 2022: 2.68 ‰, 2023: 3.0‰), in which children benefited from the high priority given to the UNHS in Luzhou and the relatively comprehensive screening network. The residents may also benefit from the stability of their lifestyle and the natural environment surrounding them. The Luzhou residents’ relatively stable lifestyle has contributed to the consistent incidence of neonatal HL associated with genetic and environmental factors. This stability in overall morbidity indirectly suggests that the natural environment and human factors in the Luzhou area have not undergone significant changes.

In the present study, the incidence of mild HL was found to be 1.07‰, accounting for 50.32% of ears with HL. Only few studies have concentrated on the prevalence of mild HL in newborns, reporting prevalence rates of 0.16–0.84 per 1,000 births [24]. The present study described the prevalence of mild HL in Chinese newborns and provided data for the investigation of mild HL. Prior to the advent of UNHS, the average age of identification of children with mild HL was 2–3 years [25]. With the popularization of UHNS, the age at the time of diagnosis of mild HL dramatically dropped to 9.6 months [26]. Furthermore, 74% of children with mild HL were identified through UHNS [27]. The incidence rates of mild HL found in this study were all higher than those reported previously [24], and the incidence of mild HL had been increasing year by year, indicating that the detection rate of mild HL was improved. This could be attributed to the continuous improvement of newborn screening technologies. With the popularization of screening technology and improvement of diagnostic level, the detection rate of mild hearing loss has increased significantly, while the distributional constituent ratio of hearing loss seems to have changed. In this study, we found that the prevalence of severe hearing loss was the lowest, which was different from the epidemiologic studies of neonatal hearing loss abroad [28], but basically consistent with the results of several domestic studies [29,30]. These findings could reflect either sampling bias or some high-risk causes that are easy to cause severe hearing loss in the sample are relatively high, requiring additional studies for verification. In the present study, it was found that 104 children were diagnosed with UHL, accounting for 36.36% of HL cases, with an incidence of about 0.95‰. Several studies have reported that the incidence of UHL was approximately 1–2‰, and UHL could account for about one-third of all children with neonatal HL [31]. Studies have characterized UHL as a minor hearing impairment [32], which is not entirely accurate. In cases of severe UHL, they may have cumulative auditory experience that is less than those with mild or high-frequency HL. Consequently, the binaural hearing advantage diminishes or even disappears, impacting speech comprehension and sound source localization, particularly in noisy environments [33]. As children with UHL typically possess normal hearing on the healthy side, their speech development remains generally unaffected. However, UHL can be somewhat insidious, making it challenging for parents to monitor HL development through daily observation. Fitzpatrick et al. conducted a study involving 108 children with UHL, revealing that 42% experienced a deterioration in hearing, 17% developed BHL, and 17% exhibited profound UHL [34]. It was also evidenced that 7.5–11% of UHL progressed to BHL [35]. Several studies have shown that the prevalence of UHL increases with age [36,37]. The early detection of UHL may be an early sign of delayed or progressive HL. Therefore, children with UHL need regular checkups to detect hearing deterioration, and school-age children should be screened regularly to identify potential UHL cases.

The Joint Committee on Infant Hearing from the American Academy of Pediatrics reported several risk factors for congenital or late-onset childhood HL [6]. In the present study, 32.87% of children surveyed had an unknown risk factor, and 8.39% of cases had a family history of deafness. Several studies have shown that genetic factors are the most common etiology of neonatal HL [38–40]. Notably, due to the Family Planning Policy implemented in China for over 30 years, along with increasing educational levels and the widespread dissemination of basic legal knowledge, the incidence of consanguineous marriages has significantly decreased, leading to a notable reduction in the number of children with hereditary HL [41]. Furthermore, the large proportion of unknown causes in this study may be attributed to the limited availability of genetic testing. In Luzhou, an economically underdeveloped region in southwest China, genetic testing is not widely accessible and is expensive, placing a financial burden on parents. Meanwhile, parents did not ask for genetic testing in the absence of a family history of deafness, as they believed that genetic testing may not change the child’s hearing outcome.

This study contains some limitations that should be pointed out. Firstly, this study used a cross-sectional design, which lacked long-term follow-up and made it difficult to determine causality. Secondly, because of the late establishment of the Luzhou Sub-center for the Diagnosis and Treatment of Neonatal Hearing Impairment in Sichuan Province, hearing data prior to its establishment were unavailable for making comparison with the current situation. Thirdly, the collection of risk factors might be limited by information review, especially for factors, such as the mother’s pregnancy history, and there might be recall bias, which might influence the accuracy and completeness of the risk factors. Finally, the social, economic, and cultural characteristics of Luzhou may be different from those of other regions, and thus, the results of this study may not be broadly applicable. The geographic specificities of this region must be considered when extrapolating the study’s results to other areas.

## Conclusions

In summary, the incidence of neonatal HL in Luzhou was 2.61‰ during 2020–2023. The prevalence was higher in male children than that in female children, involving more cases of BHL than UHL. The most common degree of HL observed was mild. The analysis of risk factors for neonatal HL in this region did not identify any significant or abnormal risk factors.

## Supporting information

S1 Table 2020–2023 neonatal hearing diagnostic information. (XLSX)
